# Genome-Wide Association Analysis of Incident Coronary Heart Disease (CHD) in African Americans: A Short Report

**DOI:** 10.1371/journal.pgen.1002199

**Published:** 2011-08-04

**Authors:** Maja Barbalic, Alex P. Reiner, Chunyuan Wu, James E. Hixson, Nora Franceschini, Charles B. Eaton, Gerardo Heiss, David Couper, Thomas Mosley, Eric Boerwinkle

**Affiliations:** 1 Human Genetics Center, University of Texas Health Science Center at Houston, Houston, Texas, United States of America; 2 Department of Epidemiology, University of Washington, Seattle, Washington, United States of America; 3 Division of Public Health Sciences, Fred Hutchinson Cancer Research Center, Seattle, Washington, United States of America; 4 Department of Epidemiology, University of North Carolina, Chapel Hill, North Carolina, United States of America; 5 Center for Primary Care and Prevention, Alpert Medical School, Brown University, Providence, Rhode Island, United States of America; 6 Department of Biostatistics, University of North Carolina, Chapel Hill, North Carolina, United States of America; 7 Department of Medicine, University of Mississippi Medical Center, Jackson, Mississippi, United States of America; Georgia Institute of Technology, United States of America

## Abstract

African Americans have the highest rate of mortality due to coronary heart disease (CHD). Although multiple loci have been identified influencing CHD risk in European-Americans using a genome-wide association (GWAS) approach, no GWAS of incident CHD has been reported for African Americans. We performed a GWAS for incident CHD events collected during 19 years of follow-up in 2,905 African Americans from the Atherosclerosis Risk in Communities (ARIC) study. We identified a genome-wide significant SNP (rs1859023, MAF = 31%) located at 7q21 near the *PFTK1* gene (HR = 0.57, 95% CI 0.46 to 0.69, p = 1.86×10^−08^), which replicated in an independent sample of over 8,000 African American women from the Women's Health Initiative (WHI) (HR = 0.81, 95% CI 0.70 to 0.93, p = 0.005). *PFTK1* encodes a serine/threonine-protein kinase, PFTAIRE-1, that acts as a cyclin-dependent kinase regulating cell cycle progression and cell proliferation. This is the first finding of incident CHD locus identified by GWAS in African Americans.

## Author Summary

In the United States, African Americans are at high risk for coronary heart disease (CHD). Although environmental and social factors have a role, genetic factors also contribute to CHD risk and mortality. Research to identify genetic factors for CHD susceptibility has been carried out mostly in Europeans and European Americans and little has been done in African Americans. Genome wide association studies (GWAS) provide a means to identify susceptibility loci without any *a priori* assumptions about the functional importance of a gene. In this study, we used GWAS to identify a novel genomic region associated with incident CHD events in African Americans from the ARIC study and replicated this finding in a large sample of African American women. This region contains several genes, including *PFTK1*, that regulate cell cycle progression and cell proliferation. This is the first report of a susceptibility locus for incident CHD identified by GWAS in African Americans.

## Introduction

Coronary heart disease (CHD) is the leading cause of death worldwide [1]. In the United States, African Americans are the most vulnerable population with regard to CHD risk factors and mortality. A recent American Heart Association report showed that African Americans are twice as likely to die from a heart-related disease compared to other ethnicities [2]. The presence of multiple CHD risk factors is 50% more likely in African Americans than in the population of European ancestry. Hypertension and diabetes are more prevalent and the highest rate of obesity is found in African American women [2]. The factors underlying these disparities are not well understood. Socioeconomic status and health care accessibility play an important role [3]. However, genetic factors are known to influence the risk of CHD [4] and population differences in the frequency and effects of these genetic factors also likely have a role.

Progress in the discovery of susceptibility genes for multiple chronic diseases and their risk factors has been made possible in recent years through genome-wide association studies (GWAS); African Americans were noticeably absent from most of these studies. GWAS have the advantage of discovering new genetic variants underlying a disease without *a priori* knowledge of gene location or function. More than 20 variants for CHD have been discovered in samples of European-descent so far [5]–[11].

In this study, we took advantage of rich longitudinal data on CHD incident events in the Atherosclerosis Risk in Communities (ARIC) study and performed a GWAS of incident CHD events in African Americans. Results that reached pre-specified genome-wide significance were investigated in African Americans from the Women's Health Initiative (WHI) [12].

## Results/Discussion

Genome-wide association analysis for incident CHD was carried out in African Americans from the ARIC study. After 19 years of follow-up, 362 individuals developed CHD and 2,543 individuals were free of CHD. Descriptive statistics for multiple cardiovascular disease risk factors at the baseline examination are provided in Table 1. Three loci reached the pre-specified genome-wide significance threshold (p<5×10^−8^) (Table 2) and were considered for replication in a sample of African American women from WHI. Results are shown in Table 2. For all variants, the direction of effect was consistent in both studies, but only the variant rs1859023 reached statistical significance in WHI (Table 2). The rs1859023 minor allele A (frequency = 0.31) had a protective effect on CHD risk with a hazard ratio (HR) of 0.57 (95% CI 0.46 to 0.69, p = 1.86×10^−08^) in ARIC and 0.81 (95% CI 0.70 to 0.93, p = 0.005) in WHI. After adjustment for CHD risk factors (smoking, diabetes, LDL, BMI and hypertension), the result was only slightly less significant (HR = 0.59, 95% CI 0.48 to 0.73, p = 8.53×10−^07^ in the ARIC study). After exclusions of coronary revascularization procedures from the ARIC case definition, the association became stronger (HR 0.53, 95% CI 0.43 to 0.65, p = 2.95×10^−09^), possibly due to a more homogenous definition of CHD. The pooled hazard ratio of incident CHD for combined ARIC and WHI data was 0.72 (95% CI 0.64 to 0.81, p = 1.29×10^−08^).

**Table 1:** Baseline characteristics of the ARIC sample.

Characteristic	Incident CHD	Free of CHD	p
N	362	2,543	
Age, years	54.98 (5.69)	52.91 (5.72)	<0.0001
Women, %	51.66	65.24	<0.0001
Hypertension, % 1	71.19	51.88	<0.0001
Diabetes, % 2	31.55	16.11	<0.0001
Current smoker, %	39.89	27.15	<0.0001
Total cholesterol, mg/dL	227.44 (49.62)	213.73 (44.27)	<0.0001
HDL cholesterol, mg/dL	48.32 (13.52)	56.32 (17.56)	<0.0001
LDL cholesterol, mg/dL	152.70 (45.97)	135.89 (42.53)	<0.0001
Triglyceride, mg/dL	134.36 (78.32)	110.89 (82.02)	<0.0001
BMI, kg/m2	30.01 (5.81)	29.59 (6.05)	NS
Average follow-up time (mean and range), years	9.86 (0.01–18.77)	16.17 (0.11–19.08)	
Age at incident CHD, years	65.35 (7.35)		

**Table 2:** Association results for genome-wide significant variants associated with incident CHD in ARIC African Americans and replication results in WHI.

					ARIC			Replication – WHI		
SNP	chr	MAF		gene	HR	95% CI	p	HR	95% CI	p
rs2283524	16	0.11	G	*HS3ST2*	1.89	1.55, 2.31	4.61E-10	1.08	0.85, 1.37	0.53
rs574007	7	0.27	G	*AC017060.1*	1.61	1.37, 1.90	1.01E-08	1.02	0.84, 1.20	0.96
**rs1859023**	**7**	**0.31**	**A**	***PFTK1***	**0.57**	**0.46, 0.69**	**1.86E-08**	**0.81**	**0.70, 0.93**	**0.005**

rs1859023 was not significantly associated with any of the traditional CHD risk factors at the baseline ARIC examination (LDL, HDL, SBP, DBP and BMI, data not shown). However, average thickness of the carotid artery, a common measure of subclinical atherosclerosis, was significantly different among rs1859023 genotypes (mean difference in carotid artery thickness per copy of the minor allele β = −0.0168, SE = 0.0047, p = 3.39×10^−04^) which suggest a role for this variant in the atherosclerotic process. When including carotid artery wall thickness as a covariate in the analysis of incident CHD attenuated but did not abolish the association with rs1859023 (HR = 0.59, 95% CI 0.48 to 0.73, p = 1.58×10^−06^). We attepted to replicate this observation by testing the assocation of rs1859023 and right coronary fatty streak area (% of intimal surface area) in the PDAY study consisting of 1,452 African Americans and 1,342 European Americans but the results did not reach statistical significance using a one-sided test. In the ARIC European Americans, rs1859023 was not significantly associated with incident CHD. The smallest p-value in the region surrounding rs1859023 (±50 kb) was at rs17869240 (p = 0.01, MAF = 0.07).

**Figure 1:**
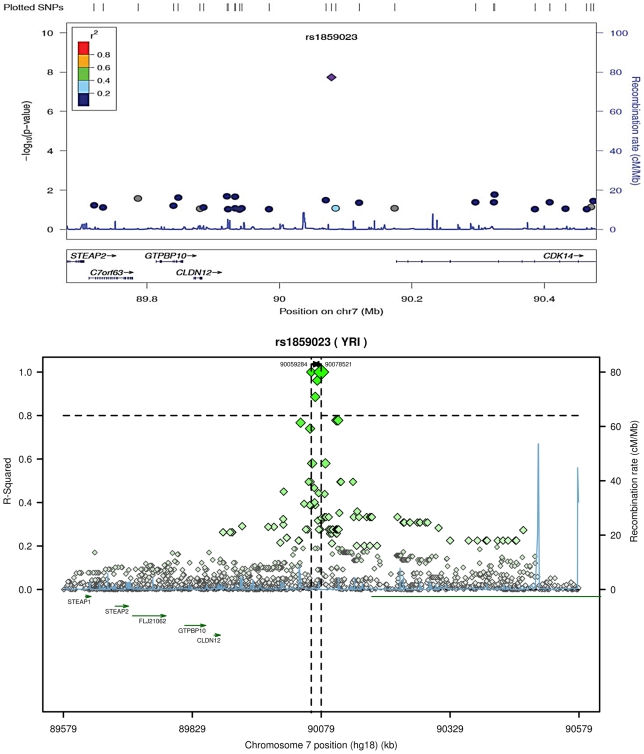
Regional plot (ARIC) of rs1859023 association with incident CHD and LD in the region arround rs1859023 (YRI) [**22]**, [23****].

rs1859023 is located in an intergenic region in proximity to the *PFTK1*, *CLDN12* and *GTPBP10* genes (Figure 1). *PFTK1*, also known as *CDK14*, encodes a serine/threonine-protein kinase PFTAIRE-1 that acts as a cyclin-dependent kinase regulating cell cycle progression and cell proliferation [13]. It is highly expressed in heart tissue [14]. In the ARIC study, rs1859023 is in loose linkage disequilibrium (LD) with other SNPs in the region (r^2^< = 0.4). In the Yoruba HapMap data, there are only 3 SNPs with LD greater than r^2^ = 0.8, all located within a very short distance of rs1859023 (Figure 1) which implies that the tagging region of rs1859023 is very narrow in African-derived populations. Given the location of the rs1859023 5′ to the *PFTK1* gene, these data imply that rs1859023 may affect gene expression. To test this hypothesis, we undertook expression QTL analyses using the resources provided at the SCAN – SNP and CNV Annotation Database (http://www.scandb.org/newinterface/about.html) [15]. rs1859023 predicts the expression of 6 genes with p-values less than 10^−4^ (Table 3). Interestingly, the evidence of rs1859023 predicting the gene expression is found only in the Yoruba population.

**Table 3:** Genes whose expression levels are associated with rs1859023.

Gene		Chr	Pop	p
*EAPP*	E2F-associated phosphoprotein	14	YRI	2E-05
*IL23A*	interleukin 23, alpha subunit p19	12	YRI	3E-05
*BCAT1*	branched chain aminotransferase 1	12	YRI	4E-05
*CLIC6*	chloride intracellular channel 6	21	YRI	4E-05
*PSCD3*	pleckstrin homology, Sec7 and coiled-coil domains 3	7	YRI	6E-05
*MTHFD2*	methylenetetrahydrofolate dehydrogenase (NADP+ dependent) 2	2	YRI	7E-05

A variant close to *PFTK1*, rs10499903, located ∼60 kb from rs1859023 was associated with ankle brachial index (ABI) in European Americans from the Framingham Heart Study (FHS) [16]. Given the different ethnic backgrounds of the two studies, and expected difference of LD patterns within the region between two samples, the Framingham result strengthens our finding and further suggests a role for the *PFTK1* gene region in the atherosclerotic process.

Some of European-discovered CHD genes have been reported to also influence CHD in African Americans [17]. However, this is the first reported GWAS finding of a CHD risk locus in African Americans. Although the sample size is less than contemporary GWAS publications in European-Americans (i.e. often exceeding 100,000 individuals (e.g. [18]), we have combined all of the available well-powered incident CHD data in African Americans with genotype data and are able to present results based on 133,415 person-years of follow-up. In addition, supporting evidence is provided by the association with subclinical atherosclerosis and expression QTL analyses. In conclusion, we have identified a region near the *PFTK1* gene as being associated with incident CHD and subclinical atherosclerosis in African Americans. Further studies are needed to examine the cellular or metabolic mechanisms underlying this association, and large population-based studies of minority populations are necessary to more fully understand the impact of genetic factors on multiple phenotypes in those that bear a disproportionate burden of disease.

## Materials and Methods

The ARIC (Atherosclerosis Risk in Communities) study is a population-based prospective cohort study of cardiovascular disease and its risk factors [19]. ARIC includes 15,792 persons aged 45–64 years at baseline (1987–89), randomly chosen from four US communities. Of these individuals, 4,266 are self-reported African Americans. Cohort members completed four clinic examinations, conducted approximately three years apart between 1987 and 1998, and followed with annual phone interviews since 1987.

Incident CHD in ARIC was ascertained by contacting participants annually, identifying hospitalizations and deaths during the prior year, and by surveying discharge lists from local hospitals and death certificates from state vital statistics offices for potential cardiovascular events [19]. A CHD event was defined as a validated definite or probable hospitalized MI, a definite CHD death, an unrecognized MI defined by ARIC ECG readings, or coronary revascularization. Participants were excluded from analyses if they had a positive or unknown history of prevalent stroke, transient ischemic attack/stroke symptoms, or CHD at the initial visit and/or being of non–African American ethnicity. Real-time, B-mode ultrasound was used to evaluate the carotid arterial intima-media wall thickness as an indicator of atherosclerosis in the ARIC study and the detailed description of its measurement is described elsewhere [17].

Genotyping was done in 15,020 ARIC participants using the Affymetrix Genome-Wide Human SNP Array 6.0. A total of 3,182 individuals remained after excluding individuals of non African American ethnicity, subjects who did not consent DNA use, unintentional duplicates with higher missing genotype rates, suspected mixed/contaminated samples, scans from one problem plate, samples with a mismatch between called and phenotypic sex, samples with genotype mismatch with 39 previously genotyped SNPs, suspected first-degree relative of an included individual, and genetic outliers based on average IBS statistics and principal components analysis using EIGENSTRAT. SNPs were excluded due to having no chromosome location, being monomorphic, having a call rate <95% and HWE-p<10^−5^. In this analysis, we considered only variants with a MAF greater than 10%.

Cox proportional hazards models with adjustment for age, gender and the first three principal components derived from EIGENSTRAT were used to estimate CHD hazard rate ratios (HRs) over a 19-year period (362 cases) under an additive genetic model. These analyses were done using PLINK and an R application for survival regression analyses. We define as “genome-wide significant” all associations with p<5×10^−8^. We define replication to be a significant (p<0.05) and directionally consistent association in an independent sample.

The WHI has two major components: (1) a clinical trial that enrolled and randomized 68,132 women ages 50–79 into at least one of three clinical trials; and (2) an observational study that enrolled 93,676 women ages 50–79 into a parallel prospective cohort study [12]. WHI participants were recruited from 1993–1998 at 40 clinical centers across the U.S. During follow-up, incident CHD events were adjudicated locally and centrally from medical records including hospital discharge summaries, ICD-9 codes, diagnostic, laboratory, surgical, and pathology reports by trained physicians blinded to randomized intervention and exposure status [20]. In the WHI replication sample, CHD was defined as MI, coronary revascularization, hospitalized angina, or CHD death. Definite and probable nonfatal MI required overnight hospitalization and was defined according to an algorithm based on standardized criteria using cardiac pain, cardiac enzymes and troponin levels, and ECG findings. CHD death was defined as death consistent with underlying cause of CHD plus one or more of the following: hospitalization for MI within 28 days prior to death, previous angina or myocardial infarction, death due to a procedure related to CHD, or a death certificate consistent with underlying cause of atherosclerotic CHD.

Of a total of 26,045 (17%) women from minority groups, 8,515 self identified African American women who had consented to genetic research were eligible for the WHI GWAS project. Genotyping was performed on the Affymetrix 6.0 array. After excluding samples due to genotyping failure, cryptic relatedness, and discrepancy between genetic ancestry and self-reported race, there were 8,421 WHI African Americans. Participants were further excluded from analyses if they had a positive or unknown history of prevalent stroke, transient ischemic attack/stroke symptoms, or CHD at the initial visit. A total of 862 incident first CHD events occurred among 8,155 eligible African American women without baseline CHD (Table 4). The mean age at study entry was 61.6+/−7.0 years (range 50–79). The mean baseline age of the cases was 64.2+/−7.2, and the mean baseline age of the non-cases was 61.3+/−6.9. The mean time to CHD event was 5.29+/−3.19 years. The mean age at CHD event was 69.5+/−7.5 years.

**Table 4:** Baseline characteristics of the WHI sample.

Characteristic	Incident CHD	Free of CHD	p
N	862	7.293	
Age, years	64.2 (7.2)	61.3 (6.9)	<0.0001
Women, %	100	100	NS
Hysterectomy, %	61.4	55.5	0.0015
WHI Hormone Trial participant, %	24.9	21.4	0.017
Hypertension, %	70.8	53.4	<0.0001
Diabetes, %	32.5	11.8	<0.0001
Current smoker, %	15.7	11.2	<0.0001
High cholesterol, %	25.9	14.7	<0.0001
BMI, kg/m2	31.9 (6.4)	30.9 (6.4)	<0.0001

This study was approved by the participating institutional IRBs, and all ARIC and WHI participants provided written informed consent, involving the sharing of data with the scientific community.

The Pathobiolobical Determinants of Atherosclerosis in Youth (PDAY) study is composed of subjects who were 15 to 34 years of age when they died of non-CVD related causes (accidents, homicides or suicides). The purpose of PDAY was to evaluate early development of atherosclerosis [21]. For this replication analysis, we genotyped rs1859023 in 2,794 individuals from PDAY - 1,452 African Americans and 1,342 European Americans and tested the association with fatty streak area in the right coronary artery.
